# Influence of the Cultivation Conditions of the Glioblastoma Neurosphere on the Expression of MALAT1 and LINCROR Long Non-coding RNA Genes

**DOI:** 10.1134/S1607672922700053

**Published:** 2023-01-18

**Authors:** D. V. Mazur, A. V. Mishanova, T. F. Kovalenko, M. I. Shakhparonov, N. V. Antipova

**Affiliations:** 1 Institute of Bioorganic Chemistry named after M.M. Shemyakin and Yu.A. Ovchinnikov, Moscow, Russia; 2 Department of Biology and Biotechnology, Higher School of Economics, Moscow, Russia

**Keywords:** glioblastoma, cancer stem cells, lincROR, MALAT1

## Abstract

Glioblastoma multiforme (GBM) is the most aggressive malignant brain tumor. One of the reasons for the resistance of GBM to treatment is the extreme heterogeneity of the tumor and, in particular, the presence of cancer stem cells (CSCs) in the population of glioblastoma cells. In this work, we investigated the effect of conditions that reduce the proportion of CSCs in the GBM cell population on the levels of long noncoding RNAs (lincROR and MALAT1) involved in the formation of the phenotype of glioblastoma cancer stem cells. We have shown that culturing under conditions that cause a decrease in cell stemness (when fetal bovine serum is added to the culture medium) affected the content of these transcripts: in the cells of most of the analyzed lines, a decrease in the level of the positive stemness regulator lincROR and an increase in the content of MALAT1 were noted.

Glioblastoma multiforme (GBM) is the most aggressive malignant brain tumor. Standard treatments for GBM include radical surgical removal, radiotherapy, and chemotherapy with the alkylating drug temozolomide [1, 2]. However, despite comprehensive therapy, the average life expectancy of patients, as a rule, does not exceed 14 months from the time of diagnosis, and the prognosis remains generally unfavorable [2]. One of the reasons of GBM resistance to treatment is extreme tumor heterogeneity and, in particular, the presence of cancer stem cells (CSCs) in the general population of glioblastoma cells, which are characterized by increased resistance to chemo- and radiotherapy [3]. In this regard, the search for therapeutic agents that reduce the proportion of CSCs or promote their differentiation into more mature tumor cells is an extremely urgent task. It also seems important to study the mechanisms of CSCs pool maintenance. A number of long non-coding RNAs (lncRNAs)—non-translated transcripts 200 nucleotides or longer—can be identified among the factors regulating CSCs content in the general population of cancer cells [4]. The lncRNAs increasing the proportion of CSCs includes lincROR (Long Intergenic Non-protein Coding RNA, Regulator Of Reprogramming). This transcript serves as a positive regulator of expression of the so-called stemness genes (*SOX2*, *OCT4*, and *NANOG*) and has oncogenic functions in many cancers [5, 6]. Previously, our laboratory showed that lincROR increases the level of a key glioblastoma CSCs marker, CD133 [7], which serves not only as one of the surface markers of CSCs, but is also involved in the pathogenesis of GBM [8]. Another lncRNA influencing the level of the mentioned marker is MALAT1 (Metastasis Associated Lung Adenocarcinoma Transcript 1). MALAT1 is known as an oncogene in many cancers [9]. At the same time, there is evidence that this lncRNA can perform oncosupressor functions in GBM. For example, MALAT1 was found to contribute to the reduction of CD133 levels [10]. In accordance with these results, bioinformatic analysis of TCGA database, performed earlier in our laboratory, showed that decreased MALAT1 level correlates with shorter overall survival of GBM patients [11].

Based on the data mentioned above, it seems important to investigate the effect of various factors contributing to the reduction of the CSCs pool in the tumor on the levels of MALAT1 and lincROR. One of the approaches to reduce the content of CSCs in the total population of GBM cells in in vitro experiments is cultivation of cancer cells using medium containing fetal bovine serum (FBS) [12]. Thus, the aim of our study was to analyze the levels of MALAT1 and lincROR in GBM cells cultured in liquid DMEM F12 medium and in DMEM medium containing 10% FBS. For these experiments, we used GBM spheres lines obtained from 4 patients (011, 019, 022, and 067), which can serve as the most representative tumor models [13]. Two of these lines belonged to the most aggressive, mesenchymal subtype of GBM (022 and 067), whereas cells from lines 011 and 019 belonged to the less malignant proneuronal subtype of glioblastoma [14]. We also used a standard immortalized glioblastoma line, U87MG, which is characterized by high proliferative potential and at the same time reduced heterogeneity (compared to the original tumor) [15]. Medium A included DMEM/F12 containing 2 mM glutamine, 10% FBS, 100 U/ml penicillin, 100 mg/L streptomycin, and 1 mM sodium pyruvate. Medium B contained DMEM/F12 with 2% MACS NeuroBrew-21, 2 mM glutamine, 20 ng/mL epidermal growth factor (EGF), 20 ng/mL basic fibroblast growth factor (bFGF), 100 U/mL penicillin, 100 mg/L streptomycin and 1 mM sodium pyruvate.

When using serum-free medium, GBM cells formed spheroids (neurospheres) (Figs. 1a, 1b). At the same time, as one would expect, after cultivation on medium containing FBS, the cells changed their morphology and acquired the phenotype of more differentiated cells with a large number of branches and forming an adhesive monolayer (Figs. 1c–1e).

**Fig. 1.**
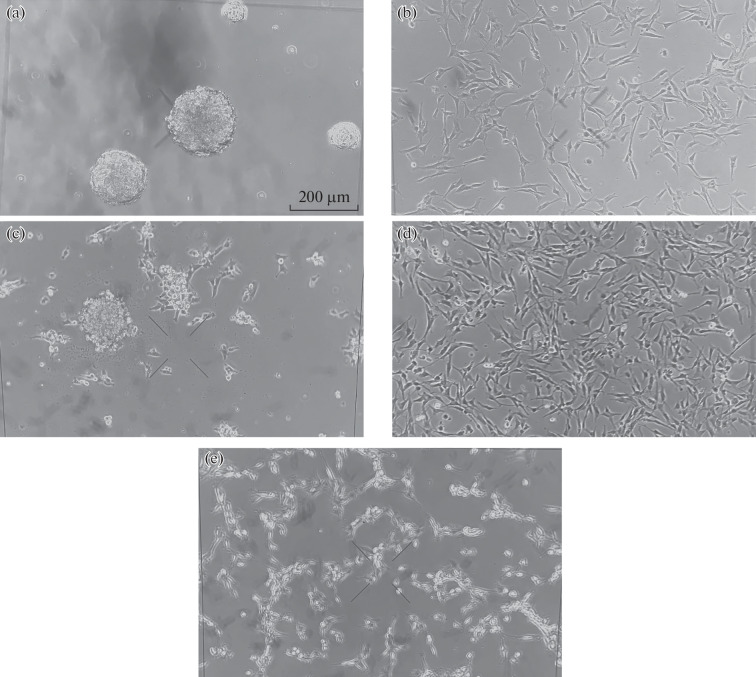
Changes in the morphology of GBM cells under different cultivation conditions. (a) 011 cells after cultivation in DMEM/F12 medium containing 2% MACS NeuroBrew-21, 2 mM glutamine, 20 ng/mL EGF, 20 ng/mL bFGF and 1 mM sodium pyruvate (medium B), (b) 067 cells after cultivation in B medium, (c) 011 cells after cultivation in DMEM/F12 medium supplemented with 10% FBS, 2 mM glutamine and 1 mM sodium pyruvate (medium A), (d) 067 cells after cultivation in A medium, (e) U87MG cells cultivated in A medium.

Next, RNA was isolated from cells grown in both medium A and medium B. Then cDNA was synthesized on RNA-template and the relative levels of MALAT1 and lincROR were analyzed by quantitative PCR (Fig. 2).

**Fig. 2.**
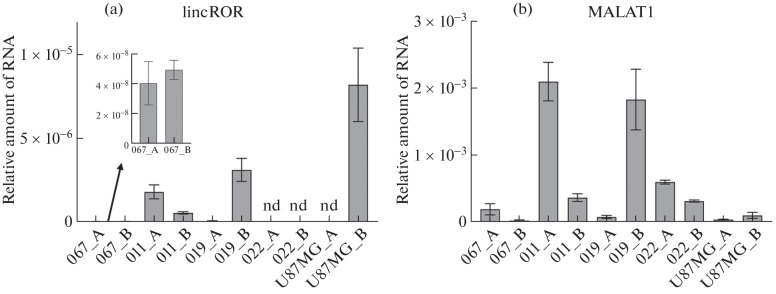
Expression of lincROR (a) and MALAT1 (b) genes in GBM cells grown on medium A or B. 18S RNA was used as an internal control.

This experiment revealed that cultivation on medium containing FBS led to a decrease in the level of lincROR (as compared to cells cultured on serum-free medium) in 3 of 5 analyzed lines: 067, 019, and U87 (in 022 cells the level of this transcript was not detected). This may indirectly indicate a decrease in the proportion of CSCs when cells of these lines are cultured under similar conditions. It is interesting to note that when cultured on medium with FBS, the level of MALAT1, on the contrary, increased in most analyzed cells. Note that mesenchymal subtype cells (022 and 067), which are characterized by high stemness, showed similar results: MALAT1 content was increased when cultured on serum-free medium. This may indicate that differentiation of CSCs under the influence of serum components may be accompanied by an increase in the level of the negative regulator of CD133 expression, MALAT1. At the same time, to our surprise, a different effect was observed for cells with the pro-neuronal phenotype (011 and 019) when cultured on medium with FBS: in patient 011 cells MALAT1 content increased, whereas in 019 cells a decrease in the level of this transcript was observed. In the case of lincROR, when cells were cultured on medium containing FBS, the level of this lncRNA increased in patient 011 cells and decreased in 019 cells. This result can be explained by the extreme heterogeneity of GBM cells: even cells belonging to the same phenotype may have different properties and respond differently to the action of different agents.

Thus, in this work, we investigated the levels of known lncRNAs (lincROR and MALAT1) in GBM cells cultured under different conditions. We showed that culturing under conditions causing a decrease in cell stemness (when FBS was added to the culture medium) affected the content of these transcripts: a decrease in the level of the positive regulator of stemness lincROR and an increase in MALAT1 content were observed in the cells of most analyzed cell lines. At the same time, we cannot but note the different effect observed in the case of 011 and 019 cells belonging to the same GBM subtype, which can be explained by the high heterogeneity of this tumor. Such facts should be taken into account both in the development of therapeutic agents and in the choice of the optimal method of treatment.
